# Postnatal depression: identification of risk factors in the short-stay maternity program in Belgium. A cross-sectional study

**DOI:** 10.3399/BJGPO.2021.0127

**Published:** 2021-11-10

**Authors:** Julie Lelièvre, Titia Hompes, Birgitte Schoenmakers

**Affiliations:** 1 University Psychiatric Centre, University Hospitals Leuven, Leuven, Belgium; 2 Department of Public Health and Primary Care, University Hospitals Leuven, Leuven, Belgium

**Keywords:** depression, postpartum, risk factors, primary health care, postnatal depression, general practice

## Abstract

**Background:**

Postnatal depression (PND) is one of the most frequent complications in women of childbearing age in the developed world. The onset of PND is influenced by several risk factors. In an attempt to avoid unnecessary long maternity stays, the Short Stay Maternity programme was launched, shifting care from the hospital environment to the outpatient setting.

**Aim:**

In order to develop an efficient programme to trace vulnerable women after childbirth and to provide support within primary care, the aim was to create an inventory of the risk factors for PND within the population of women participating in the short-stay programme.

**Design & setting:**

This study is a cross-sectional study without follow-up. Women in Belgium were invited by email to participate in the Short Stay Maternity programme within 3 months of delivery.

**Method:**

The questionnaire addressed background features and feelings during the maternity period, supplemented with the validated Dutch version of the Edinburgh Postnatal Depression Scale (EPDS). The primary outcome measure of the questionnaire was the score on the EPDS.

**Results:**

A total of 131 (27.46%) of the invited women participated. Sixteen participants (12.21%) presented with a positive score on the EPDS. The odds ratio (OR) for a positive score on the EPDS when experiencing negative feelings was 13.5 (95% confidence interval [CI] = 4.14 to 44.01). If only material support was provided, the OR for a positive EPDS score was OR 11.2 (95% CI = 2.72 to 55.5).

**Conclusion:**

In this study, two risk factors were identified for PND: negative feelings during pregnancy and the provision of only material support by the partner.

## How this fits in

Time spent in hospital after delivery is decreasing and home care has become more prominent. PND has a severe impact on both mother and child, but remains underdiagnosed. An identification of risk factors predicting PND permits proactive screening in primary care.

## Introduction

PND, comprising major depressive disorder and subthreshold depression, is one of the most frequent complications in women of childbearing age and occurs in about 10–15% of new mothers in higher income countries.^1–3^ PND is not considered a diagnostic entity by the Diagnostic and Statistical Manual of Mental Disorders, Fifth Edition (DSM-V).^4^ Major depression is defined as a depressed mood and/or anhedonia in the presence of three of the following symptoms: weight change, insomnia, energy deprivation, feeling of worthlessness, decreased concentration, and suicidality. These symptoms must be present for at least 2 weeks.^5^ During the perinatal period, women have an increased vulnerability to depression because of unavoidable psychological, biological (hormonal and immunological), and social changes.^6–8^ PND not only affects the mother, but also the child and family.^9,10^ PND can have a negative impact on the physical and mental health of the infant, resulting in physical, cognitive, and psychological delay.^11–14^ The onset of PND is influenced by the presence of several risk factors including exposure to partner violence, lack of social support, history of depression, unwanted or unplanned pregnancy, premature birth and low birth weight, maternal age at parturition (especially teenage mothers), breastfeeding, and smoking.^15–24^ Identification of risk factors helps prevention or timely detection of potential PND, and the organisation of support in a multidisciplinary context tailored to women’s needs or preferences.^25,26^

Until recently, the average postpartum hospital stay in Belgium was 5 days, which is longer than in neighbouring countries.^27^ In an attempt to avoid unnecessary long hospital stays, the Ministry of Social Affairs and Public Health proposed a general reduction of the postpartum hospital stay with a shift of postnatal and postpartum care from the hospital environment to the outpatient setting. In answer to this proposal, a consortium of hospitals and primary care organisations designed the Short Stay Maternity programme. The maternity stay of women with a low-risk pregnancy was reduced to 72 hours after delivery.^27^ This shift in care raised many additional questions about the physical and psychological wellbeing and support of mothers in the early postpartum period. It was apparent that the early detection and follow-up of patients at risk for PND deserved more attention in primary care, as negative feelings in postpartum women often remain under the radar.^28^ However, guidelines disagree on the screening strategy and on the impact and efficacy of early detection.^29^ The purposeful screening of vulnerable women is seen as the most efficient way to detect and follow women with suspected PND.

In order to develop an efficient programme to detect and screen vulnerable women after childbirth and to provide preventive support within primary care, the study aimed to create an inventory of the risk factors for PND within the population of women participating in the Short Stay Maternity programme.

## Method

This was a cross-sectional cohort study without follow-up. The study ran from July to December 2018 and 477 women were invited to participate within 3 months of delivery.

Patients were recruited based on participation in the programme of shortened maternity stay. The inclusion criteria for participation were: a planned vaginal delivery; a stay in maternity for a maximum of 72 hours after delivery; no medical indication for a longer maternity stay for mother and newborn; guaranteed follow-up at home; and the availability of a postpartum care plan (provision of home care during 10–14 days after delivery). The exclusion criteria were mild to severe complications in the mother or newborn, planned caesarean, and social vulnerability as estimated by the accompanying caregiver (midwife, gynaecologist, GP).

The mothers were contacted by email within 12 weeks after the birth to complete an electronic questionnaire addressing the feelings they experienced during the postpartum period. This questionnaire included the validated Dutch version of the EPDS supplemented with variables considered in the literature as possible risk factors for PND.^30–32^ The EPDS contains 10 questions and is considered a reliable and easy method for screening purposes. The scale is viewed as an effective screening tool for major and minor depression at a cut off of 9–10, but its accuracy is increased when the cut off is raised to 12–13.^33^ The following risk indicators were added: age at delivery, history of depression, history of PND, previous antidepressant treatment, presence of depressed feelings during pregnancy, marital status, experienced partner support, complications during pregnancy or after delivery, obstetric factors (natural vaginal delivery or assisted delivery: caesarean section), parity, breastfeeding, smoking, partner violence, prematurity, unwanted or unplanned pregnancy, and socioeconomic status.

The primary outcome measure of the questionnaire was the score on the EPDS. The number of PND symptoms was summed with a maximum total score of 30. This score was then transformed into a dichotomous variable with a cut off at value 13 (score ≥13) to propose a suggestive diagnosis of PND.^30,34^

To predict PND during or after pregnancy and to describe the prevalence of PND, the following independent variables were analysed: age at delivery, civil status, profession, parity, smoking, alcohol, history of depression or antidepressants, history of PND, negative feelings during pregnancy, planning of pregnancy, term of delivery, complications during pregnancy, in child or during postpartum, type of delivery, breastfeeding, experienced support of partner, abuse or violence during pregnancy.

A multivariate regression analysis was performed with the dichotomous outcome on the EPDS (cut off set at 13) as dependent variable. Final significance of χ² was fixed at *P*<0.05 and ORs with a value within the 95% CI were considered significant. All statistical operations (frequency and logistic procedure) were processed with SAS (version 9.4).

Patient participants signed an informed consent for participation in the Short Stay Maternity programme after verbal briefing by a researcher. Patients could withdraw from further participation with a simple opt-out button.

## Results

The online questionnaire was sent to 477 mothers and 131 (27.46%) women participated. Sixteen participants (12.21%) presented with a score ≥13 on the EPDS. Fifteen (11.45%) participants mentioned antecedents of depression and four (3.05%) presented with PND after an earlier pregnancy. Nineteen (14.50%) participants reported negative feelings during the last pregnancy. Only one participant reported abuse or violence during the pregnancy. Twenty-two (16.79%) participants underwent a caesarean. In two (1.53%) and three (2.29%) cases there was a child and mother complication after delivery, respectively. Twenty-three (17.56%) participants experienced a complication during the pregnancy. One hundred thirteen (86.26%) participants experienced enough support from their partner, but seven (5.34%) experienced only material or insufficient support (Table 1).

**Table 1.** Background features and risk indicators for postnatal depression (*n* = 131)

**Parameter**	***n* (%**)	*P* value χ² for equal proportions
Age ≥18 years	130 (99.24%)	<.0001
Marital status		
divorced	1 (0.76%)	<.0001
married or officially cohabiting	112 (85.50%)	
relationship	18 (13.74%)	
Profession		
worker	2 (1.53%)	<.0001
senior management	58 (44.27%)	
lower management	44 (33.59%)	
formally unemployed	7 (5.34%)	
independent	20 (15.27%)	
Parity		
first	58 (44.27%)	<.0001
second	50 (38.17%)	
third	20 (15.26%)	
fourth	2 (1.53%)	
fifth or more	1 (0.76%)	
Smoking	5 (3.82%)	<.0001
Depression history	15 (11.45%)	<.0001
Antidepressants history	11 (8.40%)	<.0001
Postnatal depression history	4 (3.05%)	<.0001
Negative feelings	19 (14.50%)	<.0001
Unplanned pregnancy	15 (11.45%)	<.0001
Complication (pregnancy)	23 (17.56%)	<.0001
Preterm delivery	14 (10.69%)	<.0001
Caesarean	22 (16.79%)	<.0001
Complication (child)	2 (1.53%)	<.0001
Complications (postpartum)	3 (2.29%)	<.0001
Breastfeeding	117 (89.31%)	<.0001
Support partner		
only emotional	3 (2.29%)	<.0001
only material	7 (5.34%)	
insufficient	7 (5.34%)	
sufficient	113 (86.26%)	
no partner	1 (0.76%)	
Abuse during pregnancy	1 (0.76%)	<.0001
Violence during pregnancy	1 (0.76%)	<.0001
EPDS positive score	16 (12.21%)	<.0001

EPDS = Edinburgh Postnatal Depression Scale

A multivariate logistic regression analysis with the score on the EPDS returned to a dichotomous variable and all the indicated risk factors as the predictors seemed not reliable and valid (model warning owing to quasi-complete separation of data points). After a process of introducing and excluding the risk indicators in the logistic model, only a model with partner support and negative feelings during the pregnancy appeared reliable and valid. The OR for a positive score on the EPDS when experiencing negative feelings was OR 13.5 (95% CI = 4.14 to 44.01). If the partner only provided material support than the OR for a positive EPDS score was OR 11.2 (95% CI = 2.72 to 55.5) (Table 2).

**Table 2.** Multivariate analysis with the dichotomised EPDS score as dependent variable (reference = score ≥13)

**Independent variable**	***P* value χ²**
Age ≥18 years	0.7081
Marital status	0.3578
Profession	0.8157
Parity	0.7393
Smoking	0.3951
Depression history	0.3277
Antidepressants history	0.0708^a^
Postnatal depression history	0.4277
Negative feelings	<.0001^b^
Unplanned pregnancy	0.0693
Complication (pregnancy)	0.5704
Preterm delivery	0.5398
Caesarean	0.8232
Complication (child)	0.5950
Complications (postpartum)	0.2584
Breastfeeding	0.5398
Support partner	0.0047^b^
Abuse during pregnancy	0.7081
Violence during pregnancy	0.7081

^a^Nearly significant at a 0.005 level. ^b^Significant at a 0.005 level.

## Discussion

### Summary

This study investigated the relationship between the development of PND (defined as a test score ≥13 on the EPDS) and predictive contextual factors for women participating in the Short Stay Maternity programme. More than one in 10 of all participants presented with PND. Women who experienced negative feelings during their pregnancy or women who experienced only material support from their partner were particularly at risk of presenting with PND within 3 months after delivery.

### Strengths and limitations

The major strength of the study is the sample size, which is larger than most studies in this field. A representative population was also recruited when considering the demographic features. Second, common and less common conditions were inventoried with a suspected impact on mental wellbeing. These conditions were well documented since they also served as quality indicators for the project.

The major limitation of the study is that participants were recruited during a nationwide implementation project. This strategy probably affected the study results since all women were included in a care pathway. It was observed that a care plan was missing for the most vulnerable women and in that case, the hospital stay was extended. In addition, the most vulnerable women were traced before delivery, based on the common screening programmes, and did not enter the short-stay programme.

However, the standard care plan only provided support the first 10–14 days after delivery. After that period, the onset of PND is still possible. The low response rate is a second limitation of the study. Women might have been missed who felt ashamed about the negative feelings or who felt too distressed to participate in a study. On the other hand, the sociodemographic features of this sub-sample were in line with the characteristics of the total sample of participants. Third, a single measure point was used without follow-up. It was considered that follow-up of symptoms adds to the exploration of an intervention effect in cases of PND, but not necessarily to profiling of women at risk.

### Comparison with existing literature

The reported prevalence of PND in the present study is in accordance with other studies.^4,28,35^ When the cut-off score on the EPDS was set on 11, another 13 women (almost 10% of the total number of participants) entered the risk zone of PND. Screening for PND might improve outcomes for mother and child, but both routine screening programmes and instruments are still subject of debate.^5,28,30,36^ With an increase of 10% of PND risk by minimally lowering the cut-off score on the EPDS, the sensitivity of this instrument should be further investigated. Other authors demonstrated that a cut-off value of 10 yielded a sensitivity of 100% and a specificity of 87%, which results in many false positives.^28^ To screen in women presenting with symptoms, a cut off of ≥13 could be defendable. In case a highly sensitive screening is desired, a cut-off score of 11 could be preferable considering that false positives also put a high emotional burden on both patient and relatives.^37^ The ideal strategy in primary care is one of a high sensitivity, but particularly targeting women at risk. Primary care provides are best placed to create an inventory of risk factors and to identify vulnerable women.

The questionnaire was sent to the participants in the first 3 months after delivery, which is considered the high-risk period for the onset of PND.^1^ Nevertheless, there is a non-negligible number of cases with an onset of PND between 3 and 6 months after delivery.^7,17,28^ These woman are at greater risk of remaining under the radar than women presenting with symptoms in the expected, more vulnerable, time span.^38^ For screening purposes, the cut-off score on the EPDS might therefore be adjusted to the time between delivery and screening. False negatives are least desired in the first months after delivery and false positive results probably do less harm in this time window. In the second term after delivery, the focus could be on woman presenting with symptoms and screen with a higher (regular) cut-off score. In this period, women are mainly followed by primary caregivers and access to primary care is certainly lower than to hospital care. Primary caregivers are also confident with the context of their patients. Therefore, it is important that these caregivers learn to recognise risk factors of PND and to timely screen and detect the first onset of symptoms.

In line with other research, two risk factors were identified for developing PND.^6,35^ First, women who experienced negative feelings during pregnancy were significantly more at risk than women who did not experience these feelings. In contrast, women who reported a history of PND were, according to this study, not more at risk of developing PND, while women taking antidepressants approached the risk zone (although not significantly). It is assumable that women in these groups are considered as high risk for PND and they might therefore be better surrounded with attention and support from professionals and relatives. On the other hand, there is still a taboo on reporting negative feelings during pregnancy and an underdiagnosis of depression should be considered in these vulnerable groups. Shame and the minimisation of symptoms by mothers and relatives may result in mothers becoming socially isolated. To reach these vulnerable mothers and offer them the support they need, perhaps the loaded term ‘depression’ should not be used and rather primary caregivers should refer to ‘experiencing stress’.^39,40^ In addition, the intake of antidepressants during a pregnancy might refer to a more unstable situation, while a history of depression might have moved to background history. Primary care providers should therefore pay particular attention to women who take antidepressants and be aware that this medication does not necessarily protect patients against PND.

A second single risk factor for PND was the presence of a partner whose support was limited to the provision of material support. As in other studies, women who experienced insufficient support were more vulnerable than women who received adequate support.^41^ The accent on the provision of material support might cover up a more profound and structural underlying problem in the relationship. Primary caregivers are in general caregivers of the family and therefore relatively well aware of the intrafamily relationships. In case of relational problems, a screening for PND might be indicated.

Sociodemographic features were not withheld as risk factors in this study. It should be considered that mapping income by profession might not be accurate enough to predict financial stability as the prevalence of material deprivation in working-class young families is increasing (https://www.statistiekvlaanderen.be/en/population-below-the-poverty-threshold-0).^17^ Most women in the present study lived together with a partner or were in a relationship. The physical absence of a partner seemed to not be a risk factor for PND. Most women probably organised themselves and provided adequate back-up measures (social network) in case of needs. Parity appeared not to be a risk factor for PND among the participants in contrast with findings in other studies where first parity is considered as a risk condition.^1^

As mentioned before, the participants were recruited from an implementation project to reduce maternity stay after delivery and were well surrounded with care. One of the inclusion criteria of the project was the availability of a postpartum care plan (during 10–14 days after delivery), implying the scheduled visit of a midwife and home maternity care, and home help on request. Social support is considered as an important protective factor.^42^ In home care organisation, particular attention should therefore be paid to the support of new mothers and their close family.

An unplanned pregnancy seemed to not be a risk factor for PND in the study. Nevertheless, with a *P*-value of 0.07, it was the closest non-significant risk factor and therefore this condition deserves attention. Unplanned does not necessary refer to unwanted, which is more likely to negatively influence mental wellbeing.^35^ In the present study, not a single woman described the pregnancy as unwanted, although it is unlikely that women would have participated if that was the case.

In the present study, there were no women reporting the use of alcohol during their pregnancy and therefore this was deleted in the final analysis. Campaigns for alcohol abstinence during pregnancy were successful in many countries.^43,44^ A handful of women smoked during pregnancy but this condition was not predicting PND.

Unplanned caesarean deliveries and mother or child complications did not affect the risk of PND. In case of postpartum or postnatal complications, women were very well supported after delivery and in their maternity period, which is a protective factor.^26,45^ The provision of (para-) medical care in Belgium is well organised and has a low entry threshold, in particular in the context of this project of shortened stay after delivery.^27^ During the project, only a very small number of readmissions of mother or child were registered. The threshold to lengthen the hospital stay in case of medicallyl suspicious conditions in mother or child was very low.

Most participants were breastfeeding but the women who chose not to breastfeed seemed to not be at particular risk of PND. The cause-consequence relationship between breastfeeding and depression is subject to debate.^10,24^ Here, it should be added that in the project of shortened stay, women were very well instructed and guided through the breastfeeding process, as this was one of the spearhead outcomes of the project. However, breastfeeding progressively lost ground in favour of bottle-feeding during the first 3 months after birth.

### Implications for research and practice

In this study, two main risk factors were identified for developing PND in women participating in the Short Stay Maternity programme: negative feelings during pregnancy; and the provision of only material support by the partner.

In primary care, these indicators are easy to screen for and to register, and add to a more effective screening for PND. Primary caregivers should be mindful of these risk factors, since they are likely to be the care professionals who are most familiar with their patients' home and family situation, and because barriers to access to primary care are low. The most commonly used screening instrument for PND is, at present, the EPDS. In further research, the cut-off score might be adjusted to best fit the objectives and to the target population of screening. Risk profiling of women at risk of PND should therefore be further investigated.

## Data

The datasets generated and/or analysed during the current study are available at this link (in Dutch): https://kuleuven-my.sharepoint.com/:x:/g/personal/birgitte_schoenmakers_kuleuven_be/Edo4z0SDbElIkRcDDNWWR38B7I-Rw6p1CIMfVBN4ThOHnQ?e=7FUmag.
